# Publisher Correction: Genetically encoded photocatalytic protein labeling enables spatially-resolved profiling of intracellular proteome

**DOI:** 10.1038/s41467-023-39911-6

**Published:** 2023-08-03

**Authors:** Fu Zheng, Chenxin Yu, Xinyue Zhou, Peng Zou

**Affiliations:** 1 College of Chemistry and Molecular Engineering, Synthetic and Functional Biomolecules Center, Beijing National Laboratory for Molecular Sciences, Key Laboratory of Bioorganic Chemistry and Molecular Engineering of Ministry of Education, Peking University, Beijing, 100871 China; 2 Academy for Advanced Interdisciplinary Studies, Peking-Tsinghua Center for Life Sciences, Peking University, Beijing, 100871 China; 3 College of Chemistry and Chemical Engineering, Lanzhou University, Lanzhou, 730000 China; 4 PKU-IDG/McGovern Institute for Brain Research, Peking University, Beijing, 100871 China; 5 Chinese Institute for Brain Research (CIBR), Beijing, 102206 China

**Keywords:** Chemical tools, Proteomics, Photocatalysis, Organelles

Correction to: *Nature Communications* 10.1038/s41467-023-38565-8, published online 23 May 2023

The original version of this Article contained an error in the order of names in the author list, which incorrectly listed the second author Chenxin Yu in the last position. This has now been corrected and the numbering of author affiliations has been updated accordingly in both the PDF and HTML versions of the Article.

