# Supplementary material for: Genetically encoded photocatalytic protein labeling enables spatially-resolved profiling of intracellular proteome
Source: Nat Commun. 2023 May 23;14:2978. doi: 10.1038/s41467-023-38565-8 (PMC10205723; doi:10.1038/s41467-023-38565-8)
Supplement: Supplementary file 1 — Supplementary Information [file 41467_2023_38565_MOESM1_ESM.pdf]

Genetically encoded photocatalytic protein labeling enables spatially-resolved profiling of intracellular proteome

Zheng *et al.*

Supplementary Information

Supplementary Table 1. List of key reagents used in this study.

Reagent	Vendor	Catalog number
DMEM	Gibco	C11995500BT
FBS	Gibco	10099141C
PBS	Solarbio	P1020-500ml
Trypsin	Gibco	25300062
Blasticidin	Selleck	S7419
Hank's buffered salt solution	Solarbio	H1025
OptiMEM	Gibco	31985062
Lipofectamine 2000	Invitrogen	11668019
PEI (Polyethylenimine, branched**)**	Sigma-Aldrich	408727-100ML
CellTiter 96® Aqueous One Solution Cell Proliferation Assay	Promega	G3580
Matrigel	Corning	356234
BL21 competent cell	Zomanbio	ZC121-1
DH5α competent cell	Zomanbio	ZC101-1
Stable3 competent cell	Zomanbio	ZC108-1
1 M Tris-HCl buffer, pH 7.5	Invitrogen	15567027
Imidazole	Aladdin	I108707-100g
Ampicillin	Inalco	1758-9314
Tryptone	Oxoid	LP0042B
Yeast extract	Oxoid	LP0021
IPTG	Inalco	1758-400
Ni-NTA beads	Qiagen	30210
Agar	Sigma	A7002-100g
Agarose	Sigma	1655021
TAE (Tris-acetate-EDTA) buffer	Beyotime	ST716
PCR Phanta Max Super-Fidelity DNA Polymerase	Vazyme	P505-d2
DNA loading buffer	Vazyme	P022-01
DL 15000 marker	Vazyme	MD103-01
DL 2000 marker	Zomanbio	ZM404-1
Dpn1	NEB	R0176L
TIANquick Maxi Purification	TIANGEN	DP205-02
Lightning cloning kit	Biodragon	BDIT0014
EndoFree Mini Plasmid Kit II	TIANGEN	DP118-02
Bovine serum albumin	Sangon	A500023-0100
Propargylamine	Accela	SY002930
Biotin-hydrazide	Macklin	B854932-25mg
Biotin-ethylacetamide	Macklin	B855100-50mg
Biotin-PEG-NH_2	Biomatrik	246702
DMSO	Concord	S6491

Protease Inhibitor	Roche	4693159001
Methanol (for mass spectrometry analysis)	Fisher chemical	A456-4
Sodium dodecyl sulfate	Solarbio	S8010
NaCl	Sigma	S3014-1KG
1M Tris-HCl (pH=7.6)	Solarbio	T1140
NP-40	Thermo	85124
Sodium deoxycholate	Sigma	30970
$CuSO_4$	Beijing Tongguang Fine Chemicals	104041
BTTAA	Click chemistry tools	1236-500
Biotin-azide	Aldrich	762024
Sodium ascorbate	Aladdin	S105024-100g
Bio-Rad Micro Bio-Spin P-30 Gel	Bio-Rad	7326223
Protein loading buffer	CWBio	CW0027S
Protein marker	Bio-Rad	1610374
TEMED	Biodee	T22500
APS	Beijing Yili Fine Chemicals	ylh067
SDS-PAGE stacking gel buffer (4x)	CWBio	CW0025S
SDS-PAGE separating gel buffer (4x)	CWBio	CW0026S
Acr-Bis 30:1	CWBio	CW0024S
Tris base	Vetec	V900483-5KG
Glycine	Vetec	V900144-5KG
Commassie Blue G250	Ourchem	71011284
Ethanol	Beijing Tongguang Fine Chemicals	678-2002
Acetic Acid	Beijing Tongguang Fine Chemicals	676-2007
Methanol (for Western blot analysis)	Beijing Tongguang Fine Chemicals	008-2011
TBST (20×)	Biorigin	BN20543-500ML
Fast Silver Stain Kit	Beyotime	P0017S
Clarity Western ECL Substrate	BIORAD	1705060
Tween-20	Macklin	T818927-500ML
DAPI	Thermo	D1306
Pierce BCA Protein Assay Kit	Thermo	23227
Streptavidin agarose resin	Thermo	20347
Biotin	TCI	B0463
DL-Dithiothreitol (DTT)	Sigma	D9163-5G
Iodoacetamide (IAA)	Sigma	I6125-5G
Triethylammonium bicarbonate buffer (TEAB)	Sigma	T7408-100ml

Urea	Sigma	U1250-1000g
Sequencing grade trypsin	Promega	V5111
Trypsin	Sigma	T1426
HCHO	Sigma	252549-25ml
$^{13}CD_2O$	Sigma	596388-1g
Sodium cyanoborohydride	Sigma	156159-10G
$NH_3 \cdot H_2O$	Aladdin	A112079
Formic Acid	Fluka	94318-50ml
Trifluoroacetic acid (TFA)	Macklin	T818782
Acetonitrile (ACN)	Fisher chemical	A998-4
TMT 10 plex Mass Tag Labeling Kits and Reagents	Thermo	90110
Pierce High pH Reverse Phase Peptide Fractionation Kit	Thermo	84868
N-hydroxysuccinimide (NHS)	J&K	117997
1-ethy-l-3-(3-dimethylaminopropyl) carbodiimide hydrochloride	Solarbio	E8170
D-biotin	Sigma	B4639
Anhydrous DMF	J&K	LSC0S67
5-bromonaphthalen-1-amine	Ark Pharm	AK-79091
tert-butyl prop-2-yn-1-ylcarbamate	Ark Pharm	AK-76551
$PdCl_2(PPh)_3$	Sigma	741248-1G
CuI	Energy Chemical	E060162
TEA	Aladdin	T103288-100ml
DCM	Aladdin	D116154-100ml
Na_2SO_4	Sigma	V900052-500G
Pd/C	Sigma-Aldrich	205699-1G
Chloroform-d	Aladdin	C109595-10×0.6ml
Dimethyl sulfoxide-D_6	Sigma-Aldrich	1312264
Methanol-D_4	Sigma-Aldrich	1362666

Supplementary Table 2. List of antibodies used in this study.

**Name**	**Vendor**	**Catalog number**
**α-**tubulin Mouse Monoclonal Antibody	Biodragon	B1052
HA-Tag Mouse Monoclonal Antibody	Biodragon	B1001
V5-Tag Mouse Monoclonal Antibody	Biodragon	B1168
Rabbit pAb to calnexin	Abcam	ab22595
TOMM20 Rabbit Antibody	Abcam	ab186735
Hsp60 Rabbit Polyclonal Antibody	Abcam	ab46798
G3BP2 Rabbit Antibody	Abcam	ab86135
Goat Anti-Mouse IgG(H+L), HRP Conjugated	Biodragon	BF03001
Goat Anti-Rabbit IgG (H+L), HRP Conjugated	Biodragon	BF03008

Goat anti-Rabbit-Alexa Fluor 488 IgG(H+L)	ThermoFisher	A-11034
Goat anti-Mouse-Alexa Fluor 647 IgG(H+L)	ThermoFisher	A-21236
Goat anti-Mouse-Alexa Fluor 568 IgG(H+L)	ThermoFisher	A-11031
Streptavidin-HRP	ThermoFisher	21124
Streptavidin-Alexa Fluor 568	ThermoFisher	S11226
Streptavidin-Alexa Fluor 647	ThermoFisher	S21374

Supplementary Table 3. List of fusion constructs used in this study.

**Mammalian expression plasmids**			
**Name**	**Vector**	**Localization**	**Features**
mito-miniSOG	pLX304	Mitochondria matrix	BstBI-mito-V5-miniSOG-NheI
mito-APEX2	pLX304	Mitochondria matrix	BstBI-mito-V5-APEX2-NheI
miniSOG-Sec61b	pLX304	ER membrane (ERM)	BstBI-V5-miniSOG-Sec61β-NheI
H2B-miniSOG	pLX304	Nucleus	BstBI-V5-H2B-miniSOG-NheI
G3BP1-miniSOG	pLX304	Stress granules	BstBI-V5-G3BP1-miniSOG-NheI
miniSOG-NES	pLX304	Cytoplasm	BstBI-V5- miniSOG-GS3-Halotag-NheI
miniSOG-KDEL	pLX304	ER lumen	BstBI-ss-HA-miniSOG-KDEL
SOPP3-KDEL	pLX304	ER lumen	BstBI-ss-HA-SOPP3-KDEL
**Bacterial expression plasmids**			
Name	Vector	Features	
miniSOG-His_6	pET21a	BamHI-miniSOG-XhoI-6X His-tag	
sortase A-His_6	pET21a	BamHI-sortaseA-XhoI-6X His-tag	

SUPPLEMENTARY METHODS

Chemical probe synthesis

Biotin-conjugated probes are synthesized following the protocol described in reference 1.

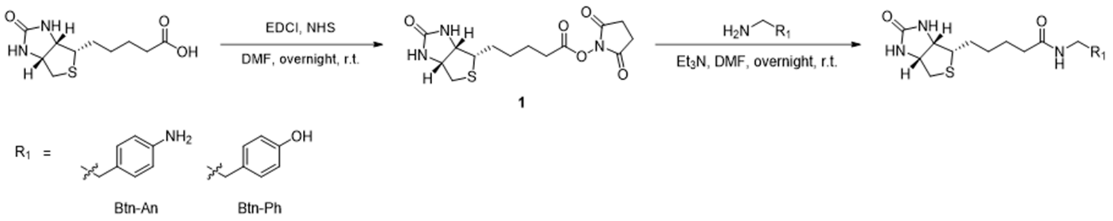

Compound 1

Compound 1 was prepared by adding *N*-hydroxysuccinimide (NHS) (4.59 g, 40.0 mmol) and 1-ethy-l-3-(3-dimethylaminopropyl) carbodiimide hydrochloride (EDC-HCl) (8.15 g, 42.6 mmol) to a solution of D-biotin (8.67 g, 35.5 mmol) in anhydrous DMF (100 mL). The reaction mixture was stirred overnight at room temperature. DMF was removed under reduced pressure to provide white solid. The white solid was washed by 4x50 mL cold ethanol and then dried under vacuum to afford compound 1. The yield was 60%.

^{1}H-NMR (400 MHz, d^6-DMSO): 6.43 (1H, s), 6.37 (1H, s), 4.30 (1H, m), 4.15 (1H, m), 3.11 (2H, m), 2.84 (1H, dd), 2.81 (4H, s), 2.67 (2H, t), 2.60 (1H, d), 1.75-1.30 (6H, m).

Biotin-Aniline (Probe 2) and Biotin-Phenol (Probe 3)

Biotin-Aniline: *N*-(4-aminophenethyl)biotinamide

Biotin-Phenol: *N*-(4-hydroxyphenethyl)biotinamide

To a solution of compound 1 (100 mg, 1.0 e.q.) and corresponding primary amine (1.0 e.q.) in 10 mL DMF was slowly added Et_3N (3.0 e.q.). The mixture was stirred overnight at room temperature. The solvent was removed by rotary evaporation. The residue was purified on a semi-preparative UPLC (Waters 2998 Photodiode Array Detector and 2545 Binary Gradient Module) equipped with a C18 reverse phase column (Waters XBridge Prep C18 5 µm OBD 19x150 mm), using a gradient of 3% to 60% methanol in water over 25 min. The overall yields were 60-70%.

^{1}H-NMR for Biotin-Aniline (400 MHz, d^6-DMSO): 7.78 (1H, t), 6.84 (2H, d), 6.49 (2H, d), 6.43 (1H, s), 6.36 (1H, s), 4.85 (2H,s), 4.31 (1H, m), 4.12 (1H, m), 3.16 (2H, dd), 3.09 (2H, dd), 2.84 (1H, dd), 2.56 (3H, m), 2.03 (2H, t), 1.38-1.66 (4H, m), 1.28 (2H, m). ^{13}C-NMR for Biotin-Aniline (100 MHz, d^6-DMSO): 25.79, 28.51, 28.65, 35.00, 35.69, 40.32, 41.07, 55.89, 59.66, 61.50, 114.42, 126.82, 129.40, 147.18, 163.18, 172.27.

^{1}H-NMR for Biotin-phenol (500 MHz, d^6-DMSO): 7.85 (1H, t), 6.96 (2H, d), 6.67 (2H, d), 6.56 (1H, s), 6.46 (1H, s), 4.30 (1H, m), 4.12 (1H, m), 3.18 (2H, dd), 3.05 (1H, dd), 2.79 (1H, dd), 2.57 (3H, m), 2.04 (2H, t), 1.42-1.61 (4H, m), 1.27 (2H, m). ^{13}C-NMR for Biotin-phenol (125 MHz, d^6-DMSO): 25.50, 28.18, 28.36, 34.56, 35.40, 40.06, 55.63, 59.46, 61.28, 115.28, 129.62, 140.70, 155.92, 163.16, 172.29, 192.48.

MS characterization of biotin probes

The following MS characterization of purified probes was acquired on Fourier Transform High Resolution Mass Spectrometry (FTMS) :

Biotin-Aniline: calculated for $C_{18}H_{27}N_4O_2S$: $[M+H]^+$: 363.18547; found: 363.18492

Biotin-Phenol: calculated for $C_{18}H_{26}N_3O_3S$: $[M+H]^+$: 364.16949; found: 364.16894

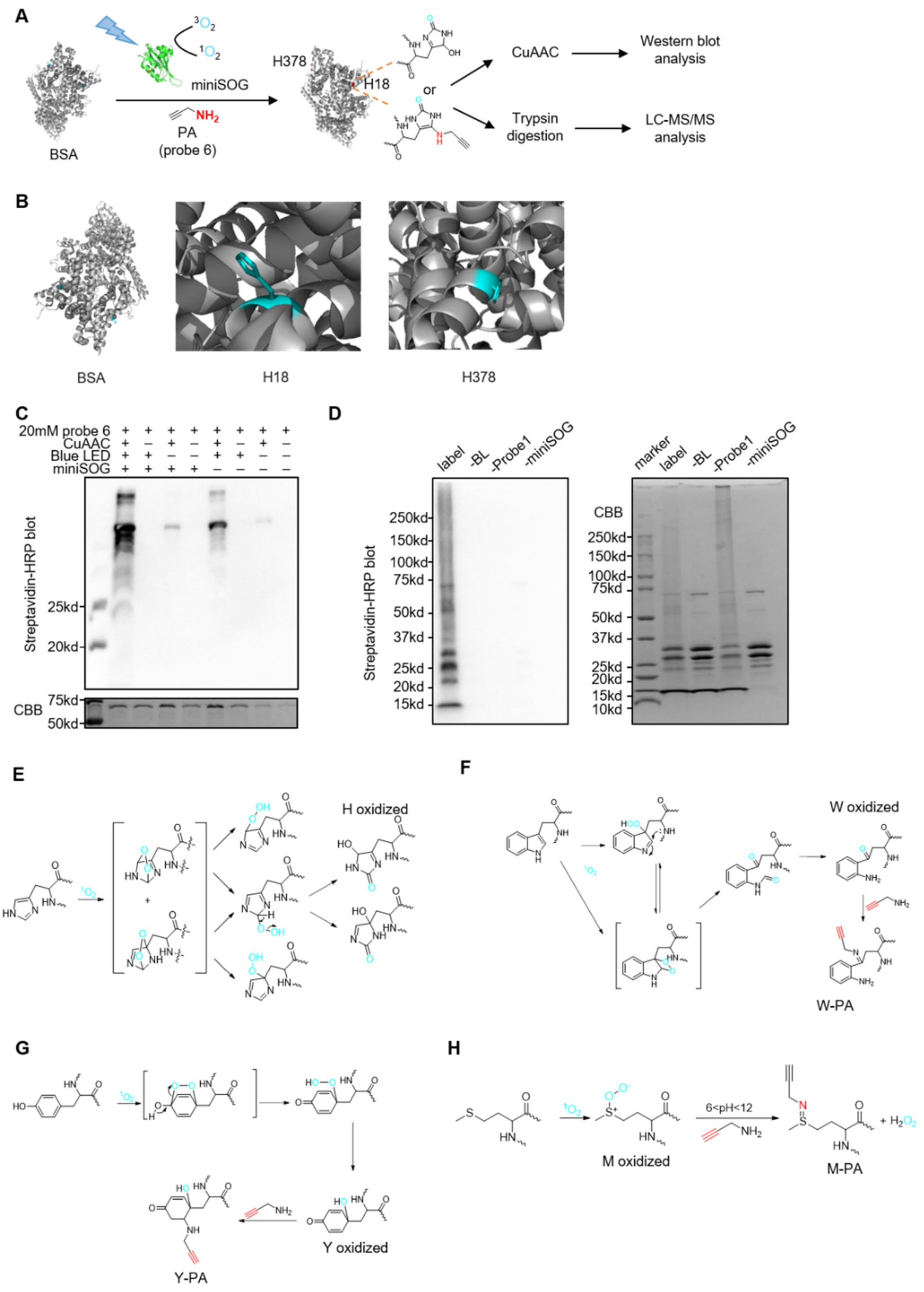

Supplementary Figure 1. miniSOG-mediated protein labeling *in vitro*. A) Scheme of BSA labeling mediated by miniSOG (PDB ID: 6GPU) and PA (probe **6)** with blue LED irradiation. **B)** Crystal structure of BSA (PDB ID: 4F5S) with two of its reactive histidine residues (H18 and H378) highlighted in cyan. Both residues are solvent-exposed. **C)** Western blot and CBB of miniSOG labeled BSA samples with controls. **D)** Western blot and CBB of miniSOG and probe 1 labeled sortase A (PDB:1T2W) samples with controls. The concentration of SDS-PAGE is 12% (C) or 4-20% gradient (D). **E)** Mechanism of histidine oxidation by singlet oxygen[4]. **F-H)** Mechanism of singlet oxygen-mediated oxidation of tryptophan, tyrosine and methionine[4] and the proposed nucleophilic addition products with probe **6**.

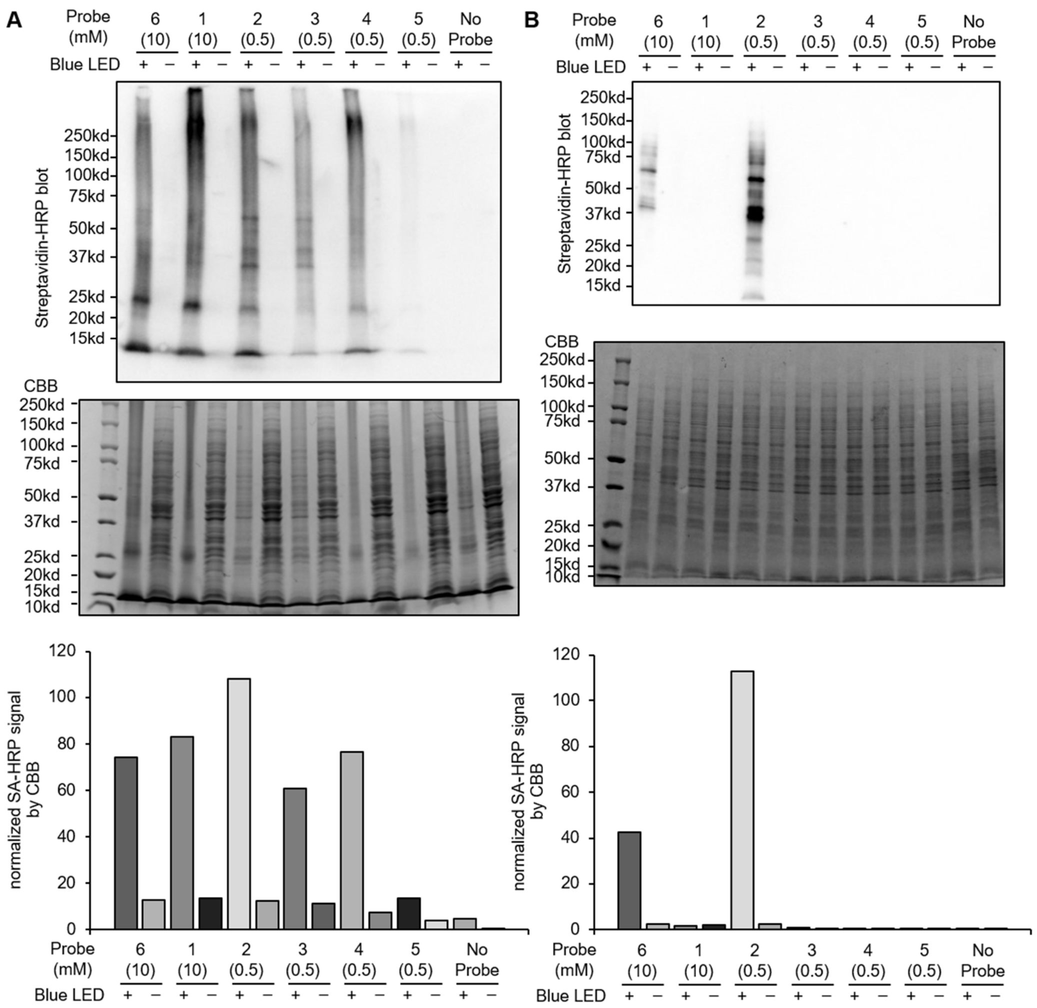

Supplementary Figure 2. Comparison of different probes for RinID activity in live cells and in cell lysates. A) Western blot and CBB of miniSOG labeled proteome samples with different nucleophilic probes in cell lysate; Probe concentration: 10 mM probe 1 and 6, 0.5 mM for other four probes. **B)** Western blot of miniSOG labeled proteome samples with different nucleophilic probes in miniSOG-NES HEK293T stable cell line. Probe concentration: 10 mM probe 1 and 6, 0.5 mM for other four probes. Bar graphs indicates the signal intensity of Streptavidin-HRP blot of each lane normalized by CBB. The concentration of SDS-PAGE is 4-20%.

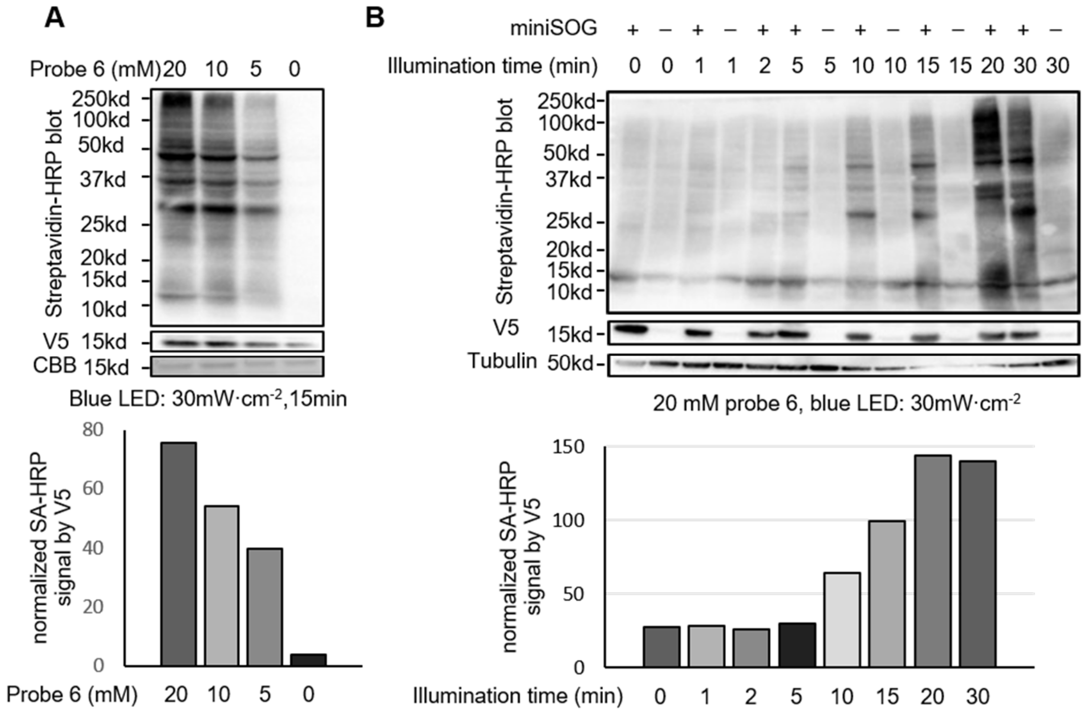

Supplementary Figure 3. Optimization of probe 6 concentration and illumination time. A-B) Western blot analysis of proteins labeled with mitochondrial matrix-targeted miniSOG (mito-V5-miniSOG) at different probe 6 concentrations (A) and illumination time (B). Bar graphs indicates the signal intensity of Streptavidin-HRP blot of each lane normalized by V5 (only miniSOG+ lanes are analyzed in (B)). The concentration of SDS-PAGE is 4-20%.

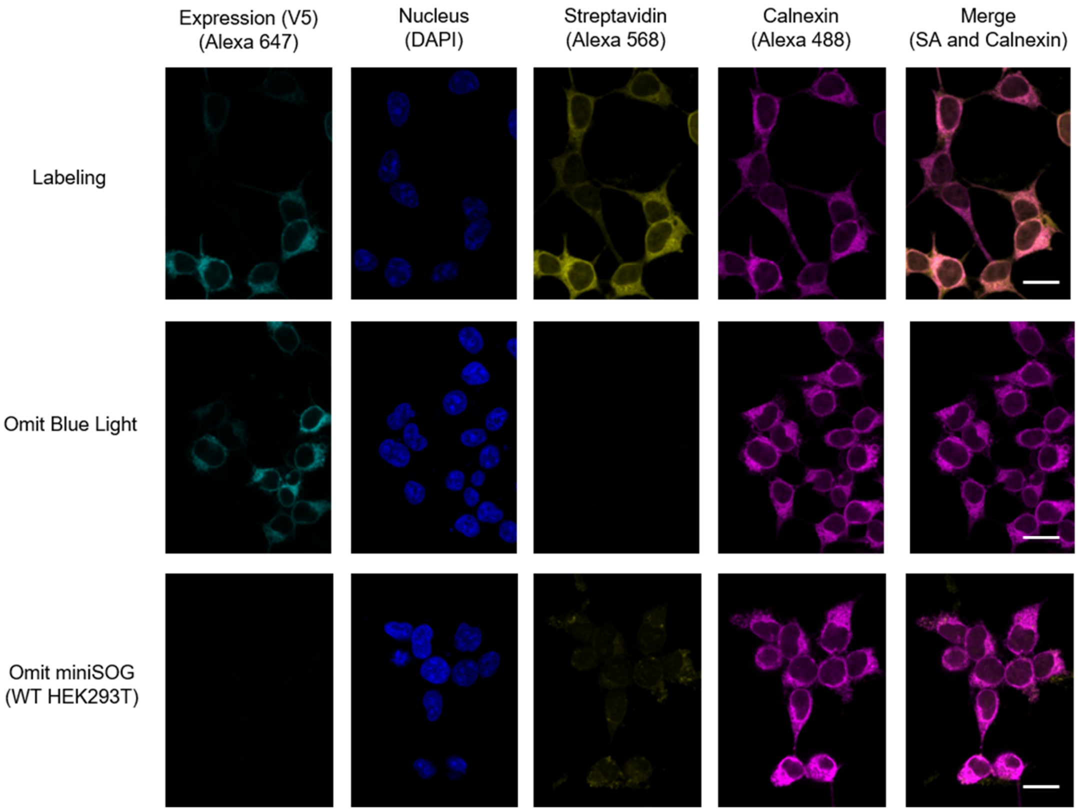

Supplementary Figure 4. Confocal fluorescence imaging of HEK293T cells expressing ERM-targeted miniSOG. Cells were labeled with 20 mM probe 6 and 30mW·cm^{-2} blue LED illumination for 15min. miniSOG was fused to V5 tag. Scale bar: 20 μm.

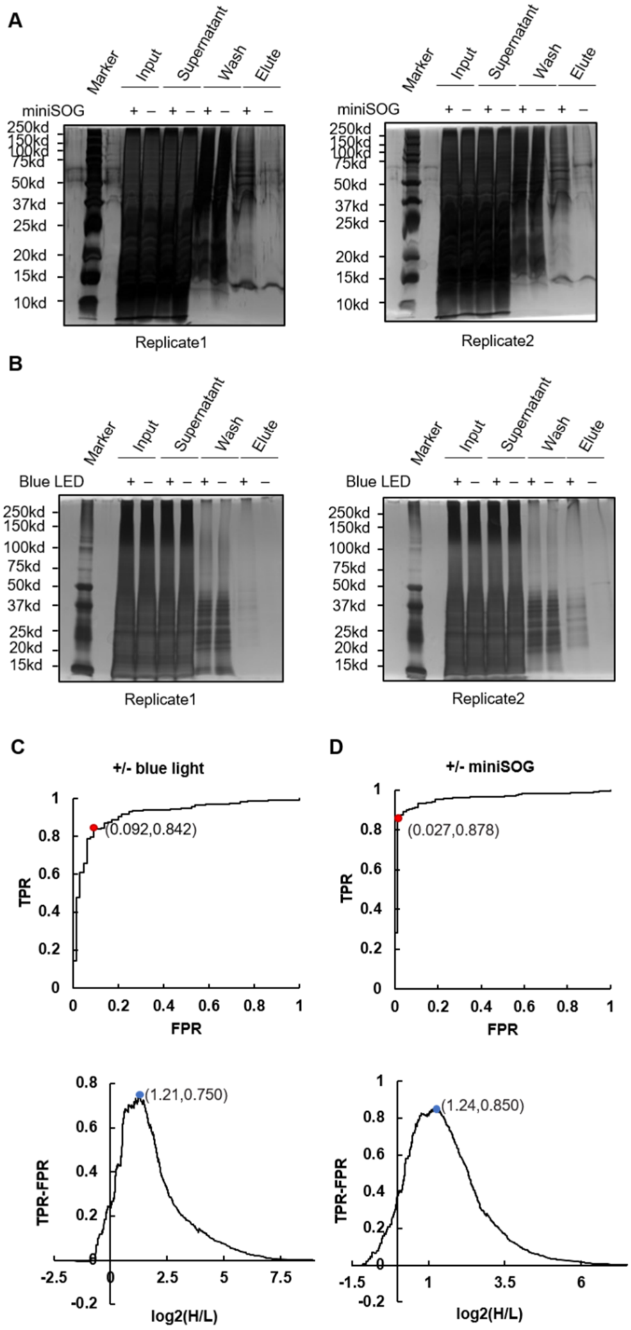

Supplementary Figure 5. Mitochondria proteome identification by RinID. A-B) SDS-PAGE and silver staining of miniSOG-labeled mitochondrial proteome samples for MS identification. Comparisons of RinID labeled sample with negative controls omitting miniSOG (A) or blue light (B). Samples were labeled with 20 mM probe 6 and 30 mW·cm^{-2} blue light illumination for 15 min. The concentration of SDS-PAGE in (A) is 12%, in (B) is 4-20%. C-D)Receiver operator curve (ROC) analysis of mitochondrial proteome identified by RinID. ROC curves of +/- blue light dataset (C) and +/- miniSOG dataset (D) are used to determine H/L cut-off . The FPR and TPR of the cut-off points are shown as red dots. The cut-off log2(H/L) and the TPR-FPR at the cut-off point are shown as blue dots.

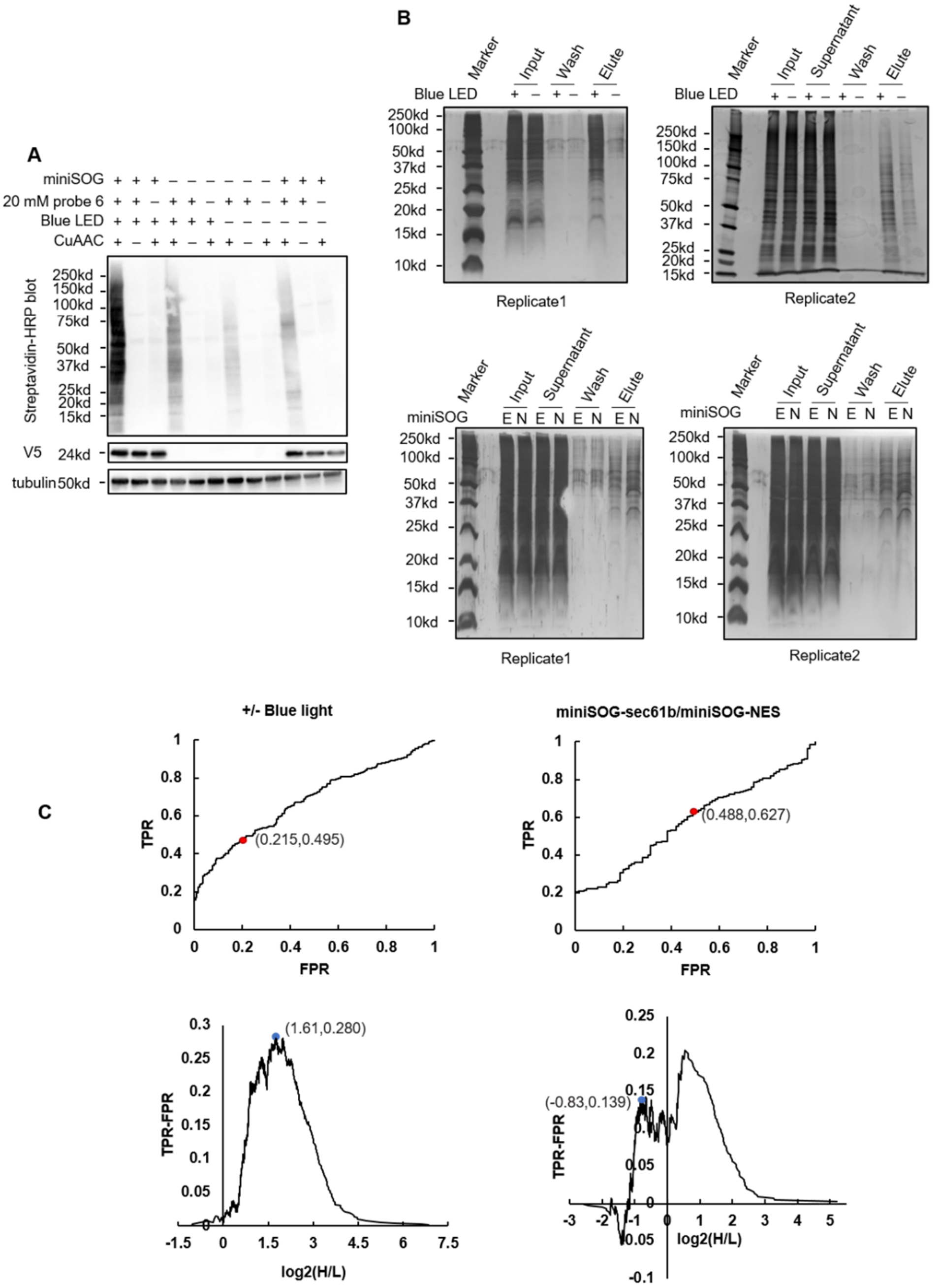

Supplementary Figure 6. ER membrane (ERM) proteome identification by RinID. A) Western blot of ERM proteome labeled by V5-miniSOG-sec61b with negative controls omitting blue light irradiation, probe 6, miniSOG, and/or CuAAC. **B)** Silver staining of ERM proteome samples labeled by V5-miniSOG-sec61b for MS identification, with negative control omitting blue light (two images on the top) or labeled with miniSOG-NES (two images at bottom). E: ERM, N: NES.The concentration of SDS-PAGE of (A) and replicate 2 of omitting blue light in (B) is 4-20%, the concentration of other three images is 12%. **C)** Receiver operator curve (ROC) analysis of ERM proteome identified by RinID. ROC curves of +/- blue light dataset (left) and ERM/NES miniSOG dataset (right) are used to determine H/L cut-off. The FPR and TPR of the cut-off points are shown as red dots. The cut-off log2(H/L) and the TPR-FPR at the cut-off point are shown as blue dots.

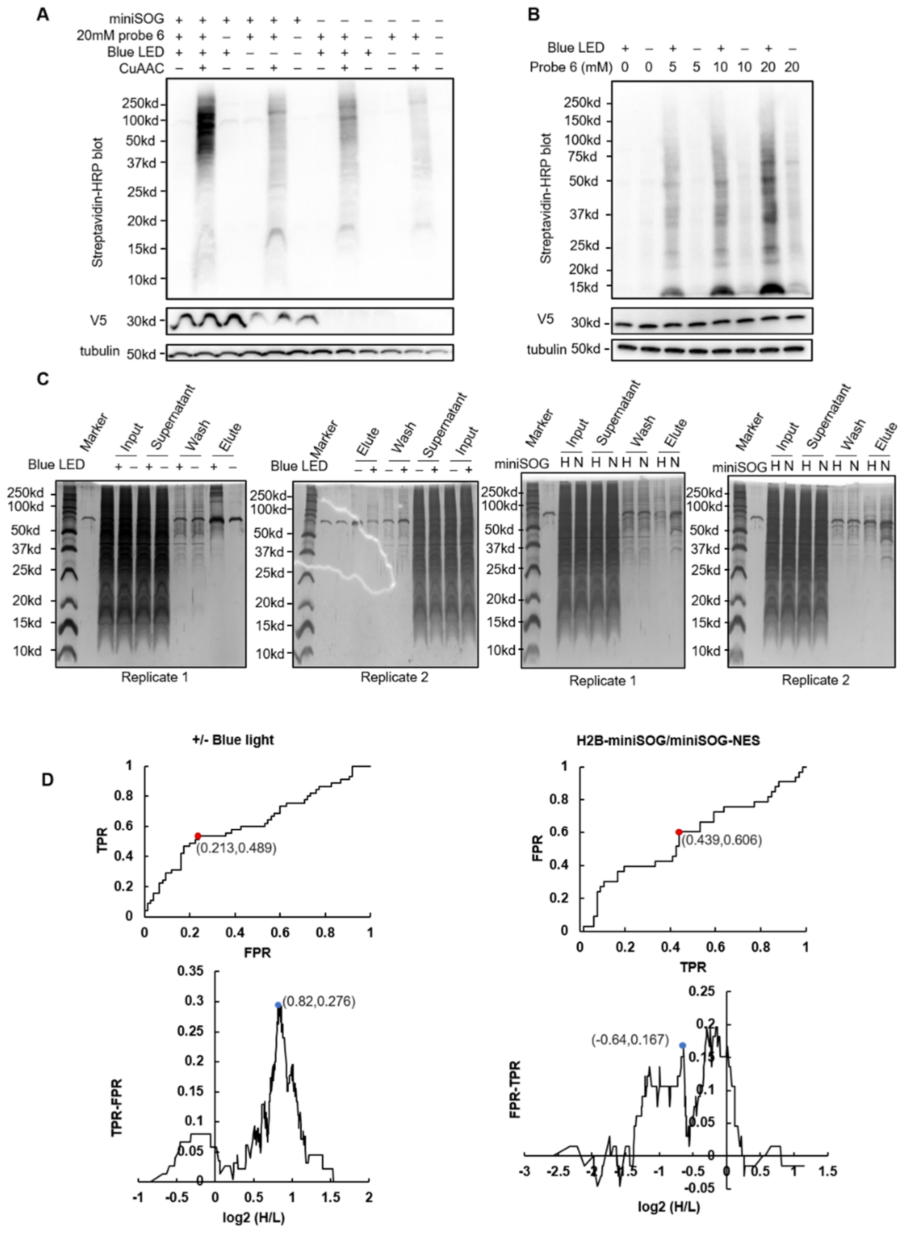

Supplementary Figure 7. Nuclear proteome identification by RinID. A) Western blot of nuclear proteome labeled by V5-H2B-miniSOG with negative controls omitting blue light irradiation, probe 6, miniSOG, and/or CuAAC. **B)** Western blot of nuclear proteome samples labeled by V5-H2B-miniSOG at different probe **6** concentrations. **C)** Silver staining of nucleus proteome samples labeled by V5-H2B-miniSOG for MS identification, with negative control omitting blue light (two images at left) or labeled with miniSOG-NES (two images at right). H: H2B; N: NES. The concentration of SDS-PAGE in (A) and (C) is 12%, in (B) is 4-20%. **D)** Receiver operator curve (ROC) analysis of nuclear proteome identified by RinID. ROC curves of +/- blue light dataset (left) and H2B/NES miniSOG dataset (right) are used to determine H/L cut-off. The FPR and TPR of the cut-off points are shown as red dots. The cut-off log2(H/L) and the TPR-FPR at the cut-off point are shown as blue dots.

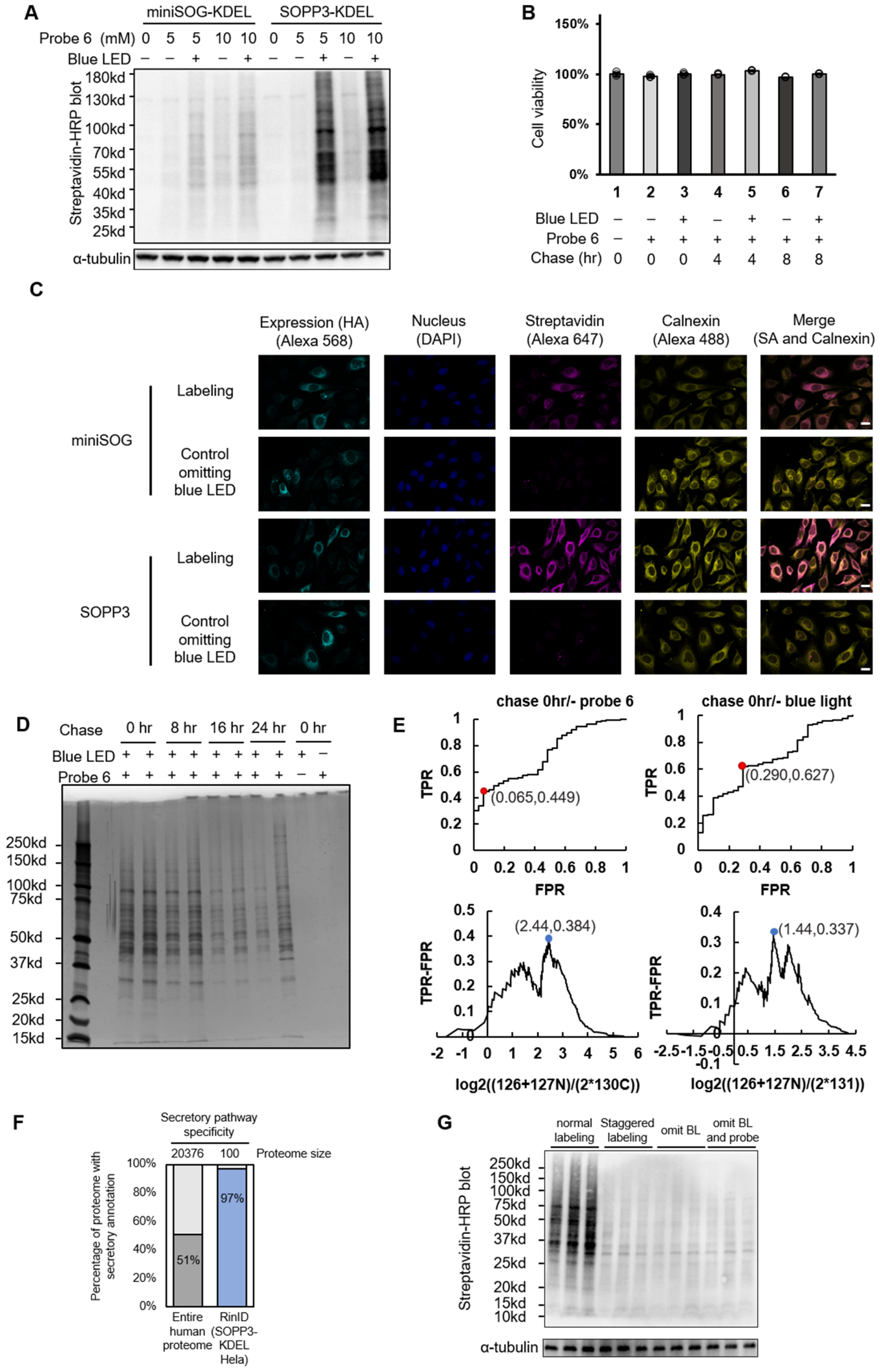
A
miniSOG-KDEL
SOPP3-KDEL
Probe 6 (mM) 0 5 5 10 10 0 5 5 10 10
Blue LED – – + – + – – + – +
Streptavidin-HRP blot
180kd
130kd
100kd
70kd
55kd
40kd
35kd
25kd
α-tubulin
B
Cell viability
150%
100%
50%
0%
1 2 3 4 5 6 7
Blue LED – – + – + – +
Probe 6 – + + + + + +
Chase (hr) 0 0 0 4 4 8 8
C
Expression (HA) (Alexa 568)
Nucleus (DAPI)
Streptavidin (Alexa 647)
Calnexin (Alexa 488)
Merge (SA and Calnexin)
miniSOG
Labeling
Control omitting blue LED
SOPP3
Labeling
Control omitting blue LED
D
Chase 0 hr 8 hr 16 hr 24 hr 0 hr
Blue LED + + + + + + + + + –
Probe 6 + + + + + + + + – +
250kd
150kd
100kd
75kd
50kd
37kd
25kd
20kd
15kd
E
chase 0hr/- probe 6
chase 0hr/- blue light
TPR
FPR
(0.065,0.449)
(0.290,0.627)
TPR-FPR
(2.44,0.384)
(1.44,0.337)
log2((126+127N)/(2*130C))
log2((126+127N)/(2*131))
F
Secretory pathway specificity
20376 100 Proteome size
Percentage of proteome with secretory annotation
100%
80%
60%
40%
20%
0%
51%
97%
Entire human proteome
RinID (SOPP3-KDEL Hela)
G
normal labeling
Staggered labeling
omit BL
omit BL and probe
Streptavidin-HRP blot
250kd
150kd
100kd
75kd
50kd
37kd
25kd
20kd
15kd
10kd
α-tubulin

Supplementary Figure 8. Pulse-chase labeling of secretory pathway proteome with SOPP3-based RinID. A) Western blot of ER lumen proteome of Hela cells labeled with miniSOG or SOPP3 at different probe 6 concentrations, with blue light illumination at 30 mW·cm^{-2} for 5 min. **B)** Cell viability assay of Hela cells at 0, 4, and 8 hours after SOPP3 labeling in the ER lumen with 5 min blue light irradiation at 30 mW·cm^{-2} and 5 mM probe 6. Error bar: mean ± SD, n = 3 biologically independent samples. **C)** Confocal fluorescence images comparing miniSOG and SOPP3 labeling in the ER lumen of Hela cells. miniSOG and SOPP3 are targeted to the ER lumen via N-terminal fusion of Igk secretory sequence and C-terminal fusion of KDEL motif (ss-miniSOG/SOPP3-KDEL). Calnexin is a marker for ER. Labeling condition: 5 mM probe 6, 30 mW·cm^{-2}, 5 min blue LED irradiation. Scale bar: 20 μm. **D)** Silver staining of ER lumen proteome samples of Hela cells pulse-chase labeled by ss-SOPP3-KDEL for MS identification, with negative control omitting probe 6 or blue light. The concentration of SDS-PAGE is 4-20%. **E)** Receiver operator curve (ROC) analysis of ER lumen proteome identified by pulse-chase RinID. ROC curves of the proteins ranked by log2((126+127N)/(2*130C)) (chase 0 hour/-probe 6) and by log2((126+127N)/(2*131)) (chase 0 hour/-blue light) are used to determine cut-off ratio. The FPR and TPR of the cut-off points are shown as red dots. The cut-off ratio and the TPR-FPR at the cut-off point are shown as blue dots. **F)** Spatial specificity analysis of ER lumen proteome identified by SOPP3 mediated pulse-chase RinID. **G)** Western blot to confirm whether probe 6 could react with the oxidized proteins after blue light irradiation. Cells were treated with four different conditions: (1) irradiated with blue LED in the presence of probe 6 in HBSS for 5 min (normal labeling); (2) irradiated with blue LED in the absence of probe 6 in HBSS for 5 min, followed by incubating with probe 6 in HBSS in the dark for 5 min (staggered labeling); (3) incubated with probe 6 in the dark in HBSS for 5 min (omit BL); and (4) incubated in HBSS in the absence of probe 6 in the dark for 5 min (omit BL and probe).

SUPPLEMENTARY REFERENCES

1. Y. Zhou, G. Wang, P. Wang, Z. Li, T. Yue, J. Wang, P. Zou, *Angew. Chem. Int. Ed.* **2019**, *58*, 11763-11767.
2. S. Rath, R. Sharma, R. Gupta, T. Ast, C. Chan, T. J. Durham, R. P. Goodman, Z. Grabarek, M. E. Haas, W. H. W. Hung, P. R. Joshi, A. A. Jourdain, S. H. Kim, A. V. Kotrys, S. S. Lam, J. G. McCoy, J. D. Meisel, M. Miranda, A. Panda, A. Patgiri, R. Rogers, S. Sadre, H. Shah, O. S. Skinner, T.-L. To, M. A. Walker, H. Wang, P. S. Ward, J., Wengrod, C.-C. Yuan, S. E. Calvo, V. K., Mootha, *Nucleic Acids Res.* **2021**, *49*, D1541-D1547.
3. Branon, T. C.; Bosch, J. A.; Sanchez, A. D.; Udeshi, N. D.; Svinkina, T.; Carr, S. A.; Feldman, J. L.; Perrimon, N.; Ting, A. Y., Efficient proximity labeling in living cells and organisms with TurboID. *Nat. Biotechnol.* **2018,** *36* (9), 880-887.
4. P. Di Mascio, G. R. Martinez, S. Miyamoto, G. E. Ronsein, M. H. G. Medeiros, J. Cadet, *Chem. Rev.* **2019**, *119*, 2043-2086.
