# Supplementary material for: Genetically encoded photocatalytic protein labeling enables spatially-resolved profiling of intracellular proteome
Source: Nat Commun. 2023 May 23;14:2978. doi: 10.1038/s41467-023-38565-8 (PMC10205723; doi:10.1038/s41467-023-38565-8)
Supplement: Supplementary file 3 — Description of Additional Supplementary Files [file 41467_2023_38565_MOESM3_ESM.docx]

File Name: Supplementary Data 1

Description: Mass spectrum data analysis of miniSOG mediated BSA *in vitro* labeling

File Name: Supplementary Data 2

Description: Mass spectrum data analysis of miniSOG mediated mitochondrial matrix proteome labeling in HEK293T cells

File Name: Supplementary Data 3

Description: Mass spectrum data analysis of miniSOG mediated ER membrane proteome labeling in HEK293T cells

File Name: Supplementary Data 4

Description: Mass spectrum data analysis of miniSOG mediated nucleus proteome labeling in HEK293T cells

File Name: Supplementary Data 5

Description: Mass spectrum data analysis of SOPP3 mediated pulse-chase ER lumen proteome labeling in HeLa cells
