# Supplementary material for: Genetically encoded photocatalytic protein labeling enables spatially-resolved profiling of intracellular proteome
Source: Nat Commun. 2023 May 23;14:2978. doi: 10.1038/s41467-023-38565-8 (PMC10205723; doi:10.1038/s41467-023-38565-8)
Supplement: Supplementary file 9 — Reporting Summary [file 41467_2023_38565_MOESM9_ESM.pdf]

nature portfolio

Corresponding author(s): Peng Zou

Last updated by author(s): Apr 25, 2023

Reporting Summary

Nature Portfolio wishes to improve the reproducibility of the work that we publish. This form provides structure for consistency and transparency in reporting. For further information on Nature Portfolio policies, see our Editorial Policies and the Editorial Policy Checklist.

Statistics

For all statistical analyses, confirm that the following items are present in the figure legend, table legend, main text, or Methods section.

n/a	Confirmed	
☐	☒	The exact sample size (n) for each experimental group/condition, given as a discrete number and unit of measurement
☐	☒	A statement on whether measurements were taken from distinct samples or whether the same sample was measured repeatedly
☐	☒	The statistical test(s) used AND whether they are one- or two-sided<br>*Only common tests should be described solely by name; describe more complex techniques in the Methods section.*
☐	☒	A description of all covariates tested
☐	☒	A description of any assumptions or corrections, such as tests of normality and adjustment for multiple comparisons
☐	☒	A full description of the statistical parameters including central tendency (e.g. means) or other basic estimates (e.g. regression coefficient) AND variation (e.g. standard deviation) or associated estimates of uncertainty (e.g. confidence intervals)
☐	☒	For null hypothesis testing, the test statistic (e.g. F, t, r) with confidence intervals, effect sizes, degrees of freedom and P value noted<br>*Give P values as exact values whenever suitable.*
☐	☒	For Bayesian analysis, information on the choice of priors and Markov chain Monte Carlo settings
☐	☒	For hierarchical and complex designs, identification of the appropriate level for tests and full reporting of outcomes
☐	☒	Estimates of effect sizes (e.g. Cohen's d, Pearson's r), indicating how they were calculated

Our web collection on statistics for biologists contains articles on many of the points above.

Software and code

Policy information about availability of computer code

Data collection	Lab View 2015; Image Lab;
Data analysis	Excel 2019; ImageJ 1.8.0; MaxQuant v1.6.10

For manuscripts utilizing custom algorithms or software that are central to the research but not yet described in published literature, software must be made available to editors and reviewers. We strongly encourage code deposition in a community repository (e.g. GitHub). See the Nature Portfolio guidelines for submitting code & software for further information.

Data

Policy information about availability of data

All manuscripts must include a data availability statement. This statement should provide the following information, where applicable:

- Accession codes, unique identifiers, or web links for publicly available datasets
- A description of any restrictions on data availability
- For clinical datasets or third party data, please ensure that the statement adheres to our policy

All MS raw data could be found on PRIDE Archive website (https://www.ebi.ac.uk/pride/)

Human research participants

Policy information about studies involving human research participants and Sex and Gender in Research.

Reporting on sex and gender	not related
Population characteristics	not related
Recruitment	not related
Ethics oversight	not related

Note that full information on the approval of the study protocol must also be provided in the manuscript.

Field-specific reporting

Please select the one below that is the best fit for your research. If you are not sure, read the appropriate sections before making your selection.

- [x] Life sciences
- [ ] Behavioural & social sciences
- [ ] Ecological, evolutionary & environmental sciences

For a reference copy of the document with all sections, see nature.com/documents/nr-reporting-summary-flat.pdf

Life sciences study design

All studies must disclose on these points even when the disclosure is negative.

Sample size	Each replicate of mitochondria proteome used one 10 cm dish of HEK293T cells for either positive or negative condition. Each replicate of ERM and nuclear proteome used two 10 cm dishes of HEK293T cells for either positive or negative condition. Each replicate of ER lumen pulse-chase labeling used two 10 cm dishes of HeLa cells for each chasing time or negative condition. Western blot used one cell of 6-wells dish of HEK293T or Hela cells for each condition. Immunofluorescence used one cell of 24-wells dish of HEK293T or Hela cells for each condition.
Data exclusions	No data were excluded.
Replication	Two biological replicates were taken for each MS experiment. Each western blot and imaging fluorescence experiments were also taken for at least 2 replicates. The replicates of each experiment showed good reproducibility.
Randomization	For the experiments taken simultaneously, the cells were from one dish of cells of the previous generation. Thus, the cells for them were randomly distributed to each condition.
Blinding	Not applicable. The experiments were designed and taken by a few of investigators and we should know the treating condition of each sample during the sample preparation.

Reporting for specific materials, systems and methods

We require information from authors about some types of materials, experimental systems and methods used in many studies. Here, indicate whether each material, system or method listed is relevant to your study. If you are not sure if a list item applies to your research, read the appropriate section before selecting a response.

Materials & experimental systems

n/a	Involved in the study	
[ ]	[x]	Antibodies
[ ]	[x]	Eukaryotic cell lines
[x]	[ ]	Palaeontology and archaeology
[x]	[ ]	Animals and other organisms
[x]	[ ]	Clinical data
[x]	[ ]	Dual use research of concern

Methods

n/a	Involved in the study	
[x]	[ ]	ChIP-seq
[x]	[ ]	Flow cytometry
[x]	[ ]	MRI-based neuroimaging

Antibodies

Antibodies used	α-tubulin Mouse Monoclonal Antibody Biodragon B1052<br>HA-Tag Mouse Monoclonal Antibody Biodragon B1001

	V5-Tag Mouse Monoclonal Antibody Biodragon B1168<br>Rabbit pAb to calnexin Abcam ab22595<br>TOMM20 Rabbit Antibody Abcam ab186735<br>Hsp60 Rabbit Polyclonal Antibody Abcam ab46798<br>G3BP2 Rabbit Antibody Abcam ab86135<br>Goat Anti-Mouse IgG(H+L), HRP Conjugated Biodragon BF03001<br>Goat Anti-Rabbit IgG (H+L), HRP Conjugated Biodragon BF03008<br>Goat anti-Rabbit-Alexa Fluor 488 IgG(H+L) ThermoFisher A-11034<br>Goat anti-Mouse-Alexa Fluor 647 IgG(H+L) ThermoFisher A-21236<br>Goat anti-Mouse-Alexa Fluor 568 IgG(H+L) ThermoFisher A-11031
Validation	All of the validation and dilutions could be found on the websites of the suppliers.

Eukaryotic cell lines

Policy information about cell lines and Sex and Gender in Research

Cell line source(s)	HEK293T cells were from American Type Culture Collection (ATCC) . HeLa cells were from National biomedical experimental cell resource bank (BMCR) of China. Stable cell lines were generated by lenti virus infection.
Authentication	Stable cell lines expressing miniSOG were confirmed by fluorescence imaging.
Mycoplasma contamination	Cell lines are PCR-tested positive for mycoplasma contamination.
Commonly misidentified lines<br>(See ICLAC register)	Not related.
